# The Opportunity of St. Bartholomew's

**Published:** 1904-10-22

**Authors:** 


### The Hospital.

A Journal of

The Medical Sciences and Hospital Administration.

Vol. XXXVII.—No. 943. OCTOBER 22, 1904.

### The Opportunity of St. Bartholomew's.

Few hospitals have been more criticised, and with reason, than St. Bartholomew's Hospital. St. Bartholomew's is our largest medical school. It is the one hospital in the country where the estates when properly administered have proved sufficient to maintain it in efficiency without appealing to the public. It has invested and other properties exceeding in value two and a half millions sterling on a low computation, and it is therefore of supreme importance that its system of administration should be as perfect as it is possible to make it. For thirty-eight years the internal administration of its affairs has been under the supreme control of a treasurer with the assistance of a resident layman called the clerk. It is under this system that it has been possible to persuade the governors to make the gravest errors, which have landed this institution in an anomalous and regrettable position. It is anomalous from the fact that, despite the great capital funds in the possession of its governors, it has appealed to the public for a large sum of money which the public has not subscribed. It is regrettable from the circumstances that, with a little prudence and foresight, this damaging blow to the reputation of our oldest hospital and medical school might and ought to have been prevented. If, however, the governors are wise they will not waste time in idle regrets, but will determine to avail themselves of the opportunity afforded by the resignation of the treasurer and the clerk to reorganise the present system of administration.

It may be well in speaking of the future to point out that, whatever changes the governors may think well to make in the existing system of management, no reflection need or ought to be cast upon anybody. The vital question for consideration is what system of administration will best secure in future the efficiency of St. Bartholomew's Hospital and promote in every way to the fullest extent the comfort and speedy recovery of its patients. We would urge this view very strongly upon the governors because it is most desirable that the problem to be solved should be approached without prejudice, and a solution found to it upon the merits alone. Both Sir Trevor Lawrence and Mr. W. H. Cross have served St. Bartholomew's Hospital to the best of their abilities, and the governors will no doubt fittingly record their sense of the value of such services.

A great general hospital with a medical school attached demands for its efficiency in the present day the establishment of a system of absolute control for every department of the institution. The greater the number of students and nurses, the larger the medical school, the greater must be the tendency to wastefulness in such matters as surgical and medical dressings and other expensive articles in daily use. Anyone who has devoted time to the inspection of hospitals in London, Scotland, and elsewhere, in proportion to the knowledge he may possess of hospital administration, must have been impressed by the evidence of efficiency and control manifest almost everywhere in those hospitals where there was a medical superintendent in charge of every department. If this efficiency be tested by figures it will be found that where the services of a medical superintendent are employed in charge of the medical department, with authority to control expenditure throughout the hospital, there the cost per bed is much less than in institutions where this system is absent. At the first blush it might be thought bad economy to add to the salaries a sum of £500 or £600 a year, or more, for a medical superintendent; but this is one of those cases where money wisely expended on salaries constitutes the most fruitful item in the expenditure of a hospital. In support of this view, in London at the present time there are three great hospitals, all equally important, one of which has a medical superintendent, whilst the other two have none. The expenditure in the first hospital, where the control of every department is efficient under its medical superintendent, is £70 a week less than in the second case, and £150 a week less than in the third. So the annual increased expenditure due to the absence of a medical superintendent in charge is £3,640 in the first, and £7,800 in the second hospital. The system here advocated has the further advantages that it secures proper control over the admission of patients, the enforcement of economy, the prevention of laxity in various ways, and at the same time tends to add immeasurably to the comfort of the patients and the staff in all departments of work.

A medical superintendent must possess high qualifications and an intimate knowledge of hospitals. Such an officer must be a man, and not a mere name. There are hospitals where such a title is affixed to an officer who exercises no real control, and is sometimes called the principal medical officer. Such a system is make-believe, and necessarily a failure. But St. Bartholomew's Hospital might well take a hint from the experience at Guy's Hospital. By appointing a medical superintendent and reorganising to the necessary extent the internal administration of its affairs St. Bartholomew's Hospital might bring about economies and attract to itself a popularity which must tend to promote the reputation and success of this great institution and all who are connected with it. It will, however, be better to attempt nothing, than merely to tinker and play with a great opportunity for reform like the present.

